# Supplementary material for: Getting More Out of Biomedical Documents with GATE's Full Lifecycle Open Source Text Analytics
Source: PLoS Comput Biol. 2013 Feb 7;9(2):e1002854. doi: 10.1371/journal.pcbi.1002854 (PMC3567135; doi:10.1371/journal.pcbi.1002854)
Supplement: Dataset S1 — GATE software. Dataset S1 bundled with this paper contains a distribution of GATE (or see http://gate.ac.uk/download/). (TGZ) [file pcbi.1002854.s001.tgz › plos-gate/src/gate/resources/gate.ac.uk/tests/tika/tika-test.doc]

# Testing Tika Format Parsers

This is a test document to ensure that we (naming no names) hasn't broken the Tika format parser again. Strangely it seems that if Tika is broken then the GATE document is created without error but with no content which is a *bit* of a pain.
